# Celiac Disease and Gluten-Free Oats: A Canadian Position Based on a Literature Review

**DOI:** 10.1155/2016/1870305

**Published:** 2016-02-24

**Authors:** Sébastien La Vieille, Olga M. Pulido, Michael Abbott, Terence B. Koerner, Samuel Godefroy

**Affiliations:** ^1^Food Directorate, Health Products and Food Branch, Health Canada, Ottawa, ON, Canada K1A 0K9; ^2^Department of Pathology and Laboratory Medicine, University of Ottawa, Ottawa, ON, Canada K1A 0L2; ^3^Département des Sciences des Aliments, Faculté des Sciences de l'Agriculture et de l'Alimentation, Université Laval, Québec, QC, Canada G1V 0A6

## Abstract

This paper provides an overview of the latest scientific data related to the safety of uncontaminated oats (<20 ppm of gluten) in the diet of individuals with celiac disease (CD). It updates the previous Health Canada position posted on the Health Canada website in 2007 and a related paper published in 2009. It considers a number of recent studies published between January 2008 and January 2015. While recognizing that a few people with celiac disease seem to be clinically intolerant to oats, this review concludes that oats uncontaminated by gluten-containing cereals (wheat, rye, and barley) can be safely ingested by most patients with celiac disease and that there is no conclusive evidence that the consumption of uncontaminated or specially produced oats containing no greater than 20 ppm gluten by patients with celiac disease should be limited to a specific daily amount. However, individuals with CD should observe a stabilization phase before introducing uncontaminated oats to the gluten-free diet (GFD). Oats uncontaminated with gluten should only be introduced after all symptoms of celiac disease have resolved and the individual has been on a GFD for a minimum of 6 months. Long-term regular medical follow-up of these patients is recommended but this is no different recommendation to celiac individuals on a GFD without oats.

## 1. Introduction

Celiac disease (CD) is a gluten-induced, immune-mediated, inflammatory process affecting almost exclusively individuals carrying HLA DQ2 and/or DQ8 [1, 2]. The prevalence of CD is about 1% in the Western world [3, 4]. People with CD react adversely to the consumption of gluten, a protein component of certain cereal grains. The relevant gluten protein fractions for people with CD include prolamins and glutenins but the alcohol-soluble fractions (prolamins) of wheat (gliadins), rye (secalins), and barley (hordeins) are considered to be of most concern to celiac individuals [5]. Oats also contain a prolamin fraction, called avenin, which is similar to gliadins, secalins, and hordeins [6]. However, oat avenins (avenae subgroup) are structurally different from the triticale prolamin fractions and represent only 10–15% of total oat protein as opposed to the prolamin content of the triticale subgroup (wheat, rye, and barley) which can be as high as 30–50% [7]. Currently, the only treatment for CD is to maintain a gluten-free diet (GFD) for life [8, 9]. For individuals with CD, careful review of food labels to determine if gluten-containing ingredients are present is essential to avoid both acute and chronic adverse health effects [10]. Accurate food ingredient lists, with no hidden sources of gluten, are important when following a GFD. The appropriate use of the term “gluten-free” on prepackaged food products helps individuals with CD to readily identify products they can safely consume. Although a GFD brings about health benefits for most people diagnosed with CD, maintaining such a diet is complex and requires a significant amount of time, effort, and commitment [10]. Moreover, the gluten-free diet is often nutritionally deficient in vitamins, calcium, iron, and fibers [11, 12]. Oats can be easily incorporated into the diet and are a good source of nutrients and fibers. However, the inclusion of oats in the GFD of people (adults and children) with CD remained controversial mainly due to a long history of cross-contamination of many oats products through normal agricultural practices with gluten sources, notably barley and wheat [13, 14]. Several long-term feeding studies have suggested that uncontaminated oats (i.e., gluten-free oats) are safe for the majority of patients with CD [15, 16]. Based on an extensive review of the scientific literature published in 2009 which included a review of the literature up to November 2008 [17], Health Canada concluded that the majority of people with CD can tolerate limited amounts of oats uncontaminated with other cereal grains such as wheat, barley, and rye. Due to limited information on long-term consumption and reports that some individuals with CD may not tolerate uncontaminated oats, the 2009 publication from Health Canada recommended that the amounts of uncontaminated oats consumed by individuals with CD should be limited to 20–25 g/day for children and 50–70 g/day for adults (http://www.hc-sc.gc.ca/fn-an/alt_formats/hpfb-dgpsa/pdf/securit/oats_cd-avoine-eng.pdf).

In order to assess the recent literature relating to CD and the safety of uncontaminated oats (containing no more than 20 ppm of gluten from other cereals), a search of scientific literature was conducted for publications on oats and celiac disease since 2008. Based on these updates, the aim of this paper is to discuss the potential use of gluten-free claims on uncontaminated-oats-based products and what limitations, if any, should be placed on the introduction of oats into the diet of individuals with CD.

## 2. Methods

### 2.1. Literature Search Strategy

A search of the available scientific literature was conducted covering the period from January 2008 to January 2015. The search was conducted in SCOPUS and PubMed databases including MEDLINE, EMBASE, and Compendex databases and Cochrane database. The following search terms were used: “celiac disease” and “oats”; “coeliac disease” and “oats”; “gluten intolerance” and “oats.” The keywords “gluten-free oats” or “uncontaminated oats” or “pure oats” but also “oat challenges” and “oats clinical trials” were used in addition to “oats.”

Study inclusion criteria were deliberately broad to reflect the difficulties involved in performing randomized controlled studies on this topic. For this reason we included randomized controlled trials, cohort studies, case control studies, cross-sectional studies, longitudinal surveys, and cross-over studies in the search. Reviews were considered separately. We included only studies on patients with biopsy-confirmed-diagnosed CD who had been exposed to oats and in whom potential changes in small-intestinal histology and in serologies and/or clinical symptoms were assessed as outcomes. Authors searched and identified potentially relevant papers after reviewing titles and abstracts of articles. Full manuscripts were obtained for those that appeared to be potentially relevant. Differences were resolved by mutual agreement. Details of the study design, aim of the study, number of subjects tested, duration of exposure to oats, diagnostic criteria used, dropout rates, and the results were extracted from each study.

### 2.2. Definition of Gluten-Free Oats

Health Canada considers that the presence of gluten at levels which do not exceed 20 ppm (parts per million) in products that are labelled “gluten-free” does not pose a risk for the vast majority of individuals who have biopsy diagnosis of CD. The choice of the 20 ppm level for the purposes of risk management is consistent with an international standard that has been established by the Codex Alimentarius Commission based on scientific premises (*Codex Alimentarius. STAN 118-1979, revised in 2008*: http://www.fao.org/fao-who-codexalimentarius/standards/list-of-standards/en/?provide=standards&orderField=fullReference&sort=asc&num1=CODEX).

A number of Western countries have already ruled on this matter and have implemented the 20 ppm level indicated in the Codex Alimentarius standard, notably the European Union, where the measure has been effective since January 2012 (Commission Regulation (EC) number 41/2009 of 20 January 2009). In August 2013, the United States Food and Drug Administration (FDA) issued a similar ruling defining the term “gluten-free” for voluntary use in the labeling of foods, stipulating that foods labelled as gluten-free cannot contain 20 ppm or more of gluten (http://www.fda.gov/Food/NewsEvents/ConstituentUpdates/ucm407867.htm). Health Canada has conducted exposure estimations to gluten from grain-containing foods and foods with grain-derived ingredients (i.e., flours), taking into consideration the various rates of food consumption by different sex and age groups in Canada. These estimates concluded that if gluten was present in foods labelled gluten-free at levels not exceeding 20 ppm, exposure to gluten would remain below 10 mg per day for all age groups studied [26]. Catassi et al. in 2007 [9] concluded that gluten exposure at levels less than 10 mg/day did not cause histological changes to the intestinal mucosa of most individuals in the study who had biopsy diagnosed CD. This finding is also supported by a review of Akobeng and Thomas in 2008 [8] which concluded that a daily gluten intake of less than 10 mg is unlikely to cause significant histological abnormalities in people with CD.

## 3. Results

Based on the review of the title and/or abstract, 33 articles and reviews were initially identified as being potentially eligible for inclusion. After reviewing the full manuscripts, 15 of these 33 articles and reviews were excluded. Among the 18 retained, 8 were reviews and 10 were original studies (5 conducted in children and 5 in adults). Clinical studies have been detailed in this section and summarised in Tables 1 and 2. Reviews were considered in Section 4.

### 3.1. Consumption of Oats and Children with CD

Table 1 summarises five clinical studies of the effects of oats on children with celiac disease published since 2008. The duration of oat exposure for these studies ranged from 6 months to 6.9 years and the amount of oats included in the gluten-free diets ranged from 3 to 50 g per day (Table 1). Two studies [19, 20] did not conduct intestinal biopsies to assess the results of the exposure to gluten. One study [20] assessed the results of the oats exposure to participants with CD based only on clinical criteria. All publications provided information verifying the purity of the oats that were used.

Based on the same Finnish cohort as Holm et al. [27], Koskinen et al. [18] studied the toxicity of oats in 23 children with CD during a 2-year follow-up. At the baseline of the study, 13 children in remission were randomized to undergo an open oats challenge and 10 children to a gluten challenge allowing the consumption of wheat, rye, and barley in addition to oats. Two children on the open oats challenge experienced abdominal pain and vomiting immediately after intake of oats and were biopsied. No signs of immune activation or relapse of CD were found but these two patients may be oat intolerant, as suggested by the authors. They concluded that the consumption of oats did not induce TTG autoantibody production at the mucosal level in children with CD as compared with the group exposed to gluten cereals.

Gatti et al. [19] administered gluten-uncontaminated oats products to 306 Italian children with biopsy-confirmed celiac disease divided into 2 groups for a 6-month period of time. Patients followed either A-B treatment (6 months of diet A, 3 months of standard GFD, and 6 months of diet B) or B-A treatment (6 months of diet B, 3 months of standard GFD, and 6 months of diet A). A and B treatments included gluten-free products with either purified oats or placebo. The addition of noncontaminated oats in one of the two groups had no impact on the clinical trend. There were no reports of dyspeptic symptoms (described in other studies as related to a high amount of fiber in oats) in this population study. Clinical symptoms were assessed by the Gastrointestinal Symptoms Rate Scale (GSRS) and the integrity of the intestinal barrier was evaluated by intestinal permeability tests (urinary lactulose/mannitol ratio). The authors concluded that the addition of noncontaminated oats in the treatment of children with CD did not cause changes in intestinal permeability and gastrointestinal symptoms.

In a retrospective study based on a food questionnaire that included 316 children and adolescents with a biopsy-confirmed CD diagnosis, Tapsas et al. [20] assessed the adverse effects of a GFD including oats. The mean time on the GFD was 6.9 years with 282 patients (90%) consuming oats in their GFD and with 38% of those doing so from the first day after being diagnosed with CD. These children were diagnosed after 2004, when the Swedish Pediatric Society recommended that oats could be included in the GFD. The other 62% were diagnosed before 2004 and changed their diets accordingly after the recommendations were launched. Most of the children (82%) ate uncontaminated oats, 45% consumed oats less than once a week, and 11% (*n* = 34) did not consume oats. Among the children who did not consume oats, 12 individuals had never tried oats, 11 had previously tried oats and did not like the taste, 2 individuals experienced symptoms (abdominal pain and loose stools) with oat consumption, and 9 did not answer the question. In this study, oats were added to the GFD for a long period and only 2 individuals had experienced symptoms, which could possibly be due to the high content of fibers in oats. The effect of a GFD with or without oats in children was published in the cornerstone study of Högberg et al. [16] where a randomised group of 93 newly diagnosed Swedish children with CD were exposed to a GFD with uncontaminated oats (25–50 g per day) or a standard GFD without oats. After 12 months, no difference in either serological markers or small-bowel mucosal architecture was observed between these two groups in this double-blind multicentre study.

As part of this study published by Högberg et al. in 2004 [16] and based on frozen samples from patients who consumed oats between 1998 and 2002, Sjöberg et al. [28] studied 28 children with symptomatic CD who were randomized into a double-blind study comparing treatment with a GFD including uncontaminated oats (GFD-oats) and a standard GFD without oats (GFD-std). Intestinal biopsies were collected from each child within 4 weeks before the study diet was introduced and after >11 months on a GFD with and without oats. There was no significant difference in serology and intestinal histology score (Marsh score) between the 2 study groups before and after the GFD intervention.

From the same study initially published by Högberg et al. [16], Tjellström et al. [29] analysed fecal short chain fatty acid (SCFA) concentrations as a marker of gut microflora metabolism. Thirty-four children from the GFD-oats group and 37 children from the GFD-std group were included in this study. Each child was studied over a period of 1 year and delivered at least one fecal sample at 0, 3, 6, and/or 12 months. In the GFD-std group, the total SCFA concentration was high at 0 and 6 months, but significantly lower after 12 months on GFD. In contrast, the total SCFA remained at a high level throughout the year for the GFD-oats group. The addition of oats to the GFD was accepted and tolerated by the majority of children studied as indicated by normalisation of the small-bowel mucosal architecture and decreasing celiac serology markers after 1-year of treatment with GFD-oats [16]. However, according to these results obtained retrospectively, the authors concluded that introduction of oats in the GFD of children with CD affected the fecal SCFA pattern considered as a marker of the gut mucosal inflammation.

### 3.2. Consumption of Oats and Adults with CD

Table 2 summarises five clinical studies of the effects of oats on adults with celiac disease published since 2008. The duration of oat exposure for these studies ranged from 3 days to 11 years. The amount of oats included in the gluten-free diets ranged from 1 to 100 g per day (Table 2). Two studies [21, 25] used serology without an intestinal mucosal biopsy to assess the consequences of exposure to gluten. Information about the purity of the oats used was not documented or not clearly defined in two of the publications [21, 23] but was provided for the others.

Guttormsen et al. [21] recruited 136 patients with a known diagnosis of CD confirmed by small-intestinal biopsy and who were following a strict gluten-free diet for at least 2 years. IgA antibodies to oat prolamins were collected from these 136 adults with treated CD and from 139 healthy individuals (controls). Among these 136 individuals, 82 had been taking oats as part of their GFD for 6 months or more and 54 did not consume oats. Of the 82 patients, 8 had increased levels of antiavenin IgA, 3 had increased levels of antigliadin IgA, and 13 had increased levels of anti-TTG IgA; the corresponding numbers among the 54 patients not exposed to oats were 4, 2, and 7, respectively. There was no statistical difference between these 2 groups of CD patients but both these groups were different from non-CD patients. The possibility that the oats being consumed were contaminated with wheat, rye, or barley was not documented in this study.

In the only available Canadian study, Sey et al. [22] investigated the safety of uncontaminated oat products manufactured under guidelines provided by the Canadian Celiac Association. Fifteen adults with biopsy-confirmed CD of >1 year duration were challenged with 350 g/week of uncontaminated oats. There were no significant changes in symptom scores, weight, hemoglobin, ferritin, and albumin among the study participants. IgA-class tissue transglutaminases antibodies remained negative in all patients and the histology scores did not change significantly during the oat challenge. The only relapse occurred in a patient who became no-compliant with her gluten-free diet.

A Finnish cross-sectional study was carried out with 177 volunteers with long-term treated CD who had been following a gluten-free diet for at least 2 years [23]. 170 out of the 177 demonstrated normal villous architecture and 7 patients had villous atrophy. Patients having normal villous architecture were split in 2 groups: 96 had persistent inflammation (intraepithelial lymphocytosis) and 74 had completely normal small-intestinal mucosa. The median duration of the GFD was 9 years in the inflammation group (*n* = 96) and 10 years in the normal group (*n* = 74). When comparing these two groups, the consumption of oats was the only factor contributing to the persistent intraepithelial lymphocytosis (comorbidities, drugs, or wheat-starch consumption had no effect). Compared to the subjects with no persistent inflammation and normal small-intestinal mucosa, the clinical outcome of the patients with persistent intraepithelial lymphocytosis was still considered good, as they had no signs of malabsorption or increase in gastrointestinal symptoms.

In a study of 106 adults, including 36 on a GFD-std and 70 consuming a GFD with oats, with a median duration of oat consumption of 5 years, Kaukinen et al. [24] concluded that daily intake and long-term consumption of oats did not result in small-bowel mucosa villous damage (assessed by small-bowel biopsies), inflammation (evaluated by IgA endomysial and IgA tissue transglutaminases antibodies), or gastrointestinal symptoms (measured by GSRS). Even long-term ingestion of oats had no harmful effects. However, two patients in this study on GFD-oats and one patient on GFD-std had abnormal villous structure on biopsies.

In Australia, 58 women and 15 men with biopsy-confirmed CD were fed a meal of oats (100 g/day for 3 days) to measure the* in vivo* polyclonal avenin specific T cell responses to peptides contained within comprehensive avenin peptide libraries [25]. In this study, 50% of patients described at least one digestive symptom following the oats challenge, but these symptoms correlated poorly with the presence of T cell responses induced by the* in vivo* challenge and with the presence of intestinal mucosal damage. The authors of the study considered that these symptoms might be explained by the large daily serving size of oats (100 g) and by the high amount of fiber in oats compared to a typical GFD. They concluded that the low rates of T cell activation after a substantial oats challenge suggest that doses of oats commonly consumed are insufficient to cause intestinal damage or serological relapse.

## 4. Discussion

*In children*, of the 5 clinical studies published during the last 7 years, 3 considered that consumption of oats which were not contaminated by gluten was safe and well tolerated for people with CD [18–20]. In two of these studies exposure to uncontaminated oats did not result in small-bowel mucosal deterioration. In the third study by Tapsas et al. [20] biopsies were not conducted. In the Italian study [19], clinical symptoms were explored by objective tools (GSRS) and laboratory parameters such as intestinal permeability tests (urinary lactulose/mannitol ratio). However, although not significantly different in the two groups studied, the number of dropouts was particularly high (36% in group A and 28% in group B) in this study. It was also not possible to analyze the data according to the amount of oats ingested which was suggested to be up to 40 g/day for older children.

For the other two studies in children, both based on the same original study published by Högberg et al. in 2004 [16], some aspects are questionable. Although there were no significant differences in antigliadin, IgA-class endomysial antibodies, and IgA-class tissue transglutaminases antibody titers and intestinal histology score (Marsh score) before and after the GFD intervention, Sjöberg et al. [28] measured expression levels of mRNAs for 22 different immune effector molecules and tight junction proteins as indicators of the immune status in the mucosa of the patients after intervention. It was found that the normalisation of genetic markers of regulators of inflammation in some pediatric patients with CD may be significantly reduced in the GFD-oats group compared to the GFD-std group (1/15 in GFD-oats group versus 6/13 in the GFD-std group). For the authors, these results suggested altered functions of the epithelium in the small intestine mucosa and supported the notion that a fraction of CD patients did not completely tolerate oats. These observations could be in line with high intraepithelial lymphocytosis counts observed in patients who had been exposed on a long-term to uncontaminated oats. However, the clinical significance of this finding is unclear since this was not associated with small-intestinal injury as evident by normal mucosa (these were the same patients as in Högberg et al. 2004 [16]). Furthermore, the methodology used to evaluate the purity of the oats being consumed was not as rigorous as the currently available ELISA methods (R5 ELISA) testing.

Tjellström et al. [29] analysed fecal short chain fatty acid (SCFA) concentrations as a marker of gut microflora metabolism and the total SCFA remained at a high level in the GFD-oats group compared to the GFD-std group. However, the SCFA fermentation index (ref. value <0.05), which mirrors intestinal inflammation, was high in both groups after 1 year of GFD. The authors reported that a GFD-std of more than one-year duration is needed to fully normalise fecal SCFA fermentation in children with CD. Another limitation was the fact that all children in the study groups did not deliver fecal samples. In addition, some of the delivered samples were too small to permit analysis.

*In adults*, 4 of the 5 studies supported the suitability of uncontaminated oats for patients with CD. The study published by Tuire et al. [23] found a persistent inflammation (intraepithelial lymphocytosis) in 56% of patients with normal villous architecture consuming oats. However, this study relied on a food consumption questionnaire and the risk of gluten contamination of oat products cannot be excluded (the oat products consumed were not confirmed to be gluten-free). In addition, the information about oat varieties consumed and the quantity of oats consumed by patients were not available for this study. Uncertainties about the purity of oats consumed can be also directed at the Norwegian study published by Guttormsen et al.; however, the authors explained that there was little reason to be concerned about adventitious presence of gluten because these oats were dedicated to a specific market and subject to rigid quality control in Norway. A final weakness of these studies was related to the number of withdrawals in some studies which were often not accurately documented, notably in the Guttormsen et al. study [21].

Overall, the different studies were relatively heterogeneous in terms of the intervention diets (amount of oats consumed per day and oat contamination), the gluten-free diet compliance, and the histological, biological, and clinical markers used. The present review was also limited by the small number of subjects (children and adults) generally enrolled in these studies, the high number of withdrawals from the studies which were often not well documented, the method of avenin isolation which was often not detailed, and data on the characteristics of oats (cultivars of oats) consumed which were not usually documented [30]. These limitations may partially explain some of the confusion over the suitability of oats for individuals with CD. Some authors considered that oat-sensitive individuals exist but noted that the occurrence of symptoms has not been associated with small-intestinal mucosal damage or inflammation [31, 32]. In a literature review, Garsed and Scott [6] supported the safe consumption of oats in the vast majority of patients with CD but considered that a small subset of patients with CD cannot tolerate oats and that some of this subset was individuals who appeared to be oats sensitive. Cooper et al. [33] in another literature review reaffirmed the lack of oats immunogenicity and toxicity to most coeliac patients. For Richman [34], there was a lack of clear evidence one way or the other about introduction of oats in the GFD. For this author, the methodology to assess potential pathology to oats was compounded by limited clinical tools of assessment. If tissue transglutaminases levels are normal, it is still possible that the small-bowel villi are damaged and of course a reduction of symptoms does not guarantee absence of small-bowel atrophy. Butzner [35] noted that relieving the restrictions on oats for patients with CD could increase the acceptability of a gluten-free diet but despite evidence supporting the safety of pure oats, there were still some individuals with CD who do not tolerate pure oats. Thies et al. [36], in the last available review of the literature, concluded that the majority of patients with CD could consume up to 100 g/day of uncontaminated oats, which would help patients adhere to a GFD.

To conclude, most authors considered that a small number of celiac patients may react adversely to oats either because they are very sensitive to the small amount of gluten contamination of their supply of oats (<20 ppm) or because they are oat sensitive.

Due to physiological mechanisms related to oat digestion and, based on the results of several studies considering that medium-high amounts (40–100 g/day) of gluten-uncontaminated oats were safely ingested for several years by most patients with CD, there is no conclusive evidence that the consumption of uncontaminated oats in patients with CD should be limited to a specific daily amount. However, even if most people with CD tolerate oats, there might be a few who have to avoid it in order to maintain remission or because they do not tolerate oats (oat-sensitive individuals). In celiac patients who experience gastrointestinal symptoms with noncontaminated oats, intestinal symptoms (dyspeptic symptoms) often occur soon after starting an oat-containing diet and can be due to an increased intake of fiber in oat products. In most cases these symptoms disappear gradually as the consumption of oats continues. If symptoms do not disappear after a couple of weeks, diagnosis of oat intolerance may be discussed.

It has also been suggested that different cultivars of oats could produce different immunological responses in people with CD. Silano et al. [37] initially observed quantitatively different toxicity of avenin from 3 oat varieties suggesting that some oat varieties maybe potentially harmful and would prevent complete mucosal recovery in individuals with CD. In another study [38], the same authors examined the immunogenicity of avenins from four oat varieties and observed that 2 varieties induced lymphocyte activation similar to that of activated wheat gliadin; in the two other oat varieties the effect was clearly lower. In a study published by Comino et al. [39], it was found that 3 groups of oat cultivars reacted differently against a specific monoclonal antibody (moAb G12 against the main immunotoxic 33-mer peptide from alpha-gliadin). One group reacted with high affinity while a second group showed slight reactivity and the last group showed no detectable reactivity suggesting that the reactivity of this antibody with cereal proteins of different variety of oats may be correlated to their immunotoxicity. Lastly, Silano et al. found significant differences among oat cultivars in eliciting the TG2-mediated events of CD inflammation [40].

These differences in immunological response to certain cultivars need to be confirmed and data on the characteristics of cultivars of oats consumed in North America should be studied in a near future.

## 5. Conclusion

Since 2008, new publications concluded that the addition of uncontaminated oats to the GFD was accepted and tolerated by the majority of CD patients, as indicated by normalisation of the small-bowel mucosal architecture and decreasing celiac serology markers. However, a few people with CD seem to be clinically intolerant to oats.

A stabilization phase should be observed before the introduction of uncontaminated oats into the GFD. Oats should only be introduced after all symptoms of CD including weight loss and growth disturbances have resolved and the individual has been on a gluten-free diet for a minimum of 6 months. In all cases, until the prevalence of oat intolerance in CD patients is established, it is recommended that all individuals with CD, including those with subclinical CD, following a GFD with oats be monitored by a physician as previously suggested by Rashid et al. [41]. The rare individual, child or adult, who develops symptoms while consuming uncontaminated oats needs to be evaluated for potential relapse of CD and for other sources of gluten contamination in their diet.

It has been suggested that only some uncontaminated oat cultivars trigger an immunological response in CD patients which could explain the chronic gut mucosal inflammation observed in some studies. The potential difference in immunotoxicity of these various oat cultivars may also explain the different clinical responses observed in patients with CD. This point needs to be confirmed and more research is required to further clarify the role of different oats cultivars in CD. Long-term regular follow-up of CD patients is still recommended for all individuals with CD and a GFD is the only available treatment; their clinical management will be the same after the introduction of oats to their diet.

This review confirms the conclusions made by Health Canada [17] on the safety of introducing uncontaminated oats into the gluten-free diet of individuals with celiac disease. More recent information suggests there is no need to restrict such consumption to a specific daily amount. Health Canada is of the position that, at levels not exceeding 20 ppm of gluten in oats as a result of cross-contamination, when Good Manufacturing Practices are followed such as the ones suggested by the Canadian Celiac Association [41], oats that are identified gluten-free, that is, labelled “gluten-free oats” as opposed to “oats” in the list of ingredients of a prepackaged food product, would not pose a health risk to most individuals with celiac disease and would meet the intent of B.24.018 (B.24.018 of the Food and Drug Regulations states that “*it is prohibited to label, package, sell or advertise a food in a manner likely to create an impression that it is a gluten-free food if the food contains any gluten protein or modified gluten protein, including any gluten protein fraction*”) of the Canadian* Food and Drug Regulations*.

## Figures and Tables

**Table 1:** Clinical studies of the effects of oats on children with celiac disease from January 2008 to January 2015.

Reference authors, year of publication	Number of subjects tested	Oats exposure	Amount of oats	Lab tests	Biopsy	Drop-out rate	Conclusion
Koskinen et al., 2009, Finland [18]	*n* = 23 13 in remission with open oats challenge; 10 in remission wheat, rye, barley, and oats	2 years	50 g/day	TTG IgA	Small-bowel biopsies (IgA deposits) at baseline + 6 m + 24 m: No signs of immune activation or relapse of CD No detrimental effect on intestinal mucosal villous morphology and IEL density	2 children experienced abdominal pain but with normal biopsy	Pure oats can be safely added to the GFD of children with CD

Gatti et al., 2013, Italy [19]	*n* = 306 Group A-B = 154 Group B-A = 152	15 months [6 months oats or placebo (A-B); 3 months washed-out; 6 months oats or placebo (B-A)]	Up to 40 g/day	TTG IgA, antigliadin IgG, and antiavenin Abs	No biopsy	55/154 (36%) enrolled in group A-B and 42/152 (28%) enrolled in group B-A No information provided about reasons for withdrawals	Addition of noncontaminated oats in the treatment of children with CD does not cause changes in intestinal permeability and gastrointestinal symptoms

Tapsas et al., 2014, Sweden [20]	*n* = 316 282 (89%) consumed oats and 259 (82%) pure oats 34 (11%) did not consumed oats	Mean = 6.9 years	Not specified	No serology	No biopsy	34 (11%) did not consume oats. For those having tried oats (*n* = 13) but stopped, 8 did not like the taste, 2 reported abdominal pain and loose stools, 2 gave no specific reason, and 1 did not answer	Most patients did not report adverse effects after long-term consumption of oats

Sjöberg et al., 2014 (Same patients as for study published in 2004 by Högberg et al. [16]), Sweden [28]	*n* = 28 15 GFD with uncontaminated oats (GFD-oats) versus 13 GFD without oats (GFD-std)	Mean = 13 months	Median = 20 g/day Range = 3–43 g/day	TTG IgA, antigliadin IgG Expression levels of mRNAs for 22 different immune effector molecules and tight junctions proteins (markers of mucosal inflammation)	No difference between 2 groups in intestinal histology score (Marsh score)		No difference between 2 groups in terms of serology biomarkers and intestinal histology score Normalisation of genetic markers of regulators of inflammation in some pediatric patients with CD maybe significantly reduced in the GFD-oats group compared to the GFD-std group Altered functions of the epithelium in the small intestine mucosa “support the notion that a fraction of CD patients tolerate oats poorly”

Tjellström et al., 2014 (Same patients as for study published in 2004 by Högberg et al. [16]), Sweden [29]	*n* = 71 34 GFD with uncontaminated oats (GFD-oats) versus 37 GFD without oats (GFD-std)	12 months	25–50 g/day	Endomysial IgA and IgG, antigliadin IgA, and TTG IgA Faecal short chain fatty acids (SCFA) concentration (marker of gut microflora metabolism) and SCFA fermentation index (marker of intestinal inflammation)	At baseline: small bowel enteropathy consistent with CD; at 12 months: all children in clinical remission except one child (GFD-std group) who did not undergo a control biopsy	None However, all children in the study did not deliver faecal samples and some samples were too small to permit analysis; distribution of missing samples was evenly distributed between the 2 study groups	Normalisation of small bowel mucosal architecture and decreasing celiac serology markers However, total SCFA remained at a high level in the GFD-oats group compared to the GFD-std group

Abs: antibodies; CD: celiac disease; GFD: gluten-free diet; IEL: intraepithelial lymphocytes; IgA: immunoglobulin A; IgG: immunoglobulin G; TTG: tissue transglutaminase; GFD-std: standard GFD without oats; GFD-oats: GFD with uncontaminated oats.

**Table 2:** Clinical studies of the effects of oats on adults with celiac disease from January 2008 to January 2015.

Reference authors, year of publication	Number of subjects tested	Oats exposure	Amount of oats	Lab tests	Biopsy	Dropout rate	Conclusion
Guttormsen et al., 2008, Norway [21]	*n* = 136 82 subjects GFD with oat consumption 54 subjects GFD without oats	2 years	Mean = 24 g/day (minimum 6 months)	Antiavenin IgA, anti-gliadin IgA, and TTG IgA	No biopsy	Not specified	Most adult CD patients can tolerate oats Ingestion of oats does not cause increased levels of IgA oats in CD patients with GFD Purity of oats not verified

Sey et al., 2011, Canada [22]	*n* = 15	3 months	50 g/day	TTG IgA	Duodenal biopsies before and after oat challenge (Marsh score): histology scores did not significantly change during oat challenge	One relapse occurred in a patient who became noncompliant with GFD	IgA TTG antibodies remained negative in all patients and the histology scores did not change. Support the safety of uncontaminated oats for patients with CD

Tuire et al., 2012, Finland [23]	*n* = 177 96 (54%) with persistent IEL 74 (42%) normal small intestinal mucosa 7 (4%) villous atrophy	Mean = 11 years	Not specified	Endomysial IgA and TTG IgA	Consumption of oats was the only factor contributing to the persistent IEL		Despite excellent villous recovery, persistent IEL was common among CD patients on a long-term GFD. Consumption of oats was associated with persistent IEL Purity of oats not verified.

Kaukinen et al., 2013, Finland [24]	*n* = 106 36 no oat in GFD 70 oats in GFD	Median = 5 years Range = 0.5–8 years	Median = 20 g/day Range = 1–100 g/day	Endomysial IgA and TTG IgA	Small-bowel mucosal biopsies were normal in 103 patients 2 patients using oats and 1 patient not using oats had abnormal structure	None	Long-term consumption of pure oats proved to be safe for CD patients. Long-term regular follow-up is recommended

Hardy et al., 2015 [25]	*n* = 73 (3 sources of oats assessed)	3 days	100 g/day	*In vivo* avenin-specific T cells responses	No biopsy	4 subjects did not complete the full 3-day challenge	34 patients reported no symptoms and 46 reported digestive symptoms. Ingestion of 100 g of oats provides weak antigenic stimulation of blood avenin-specific T cells (<10% of CD patients) which is different from the antigenic stimulation observed with wheat, rye, and barley

Abs: antibodies; CD: celiac disease; GFD: gluten-free diet; IEL: intraepithelial lymphocytes; IgA: immunoglobulin A; IgG: immunoglobulin G; TTG: tissue transglutaminase; GFD-std: standard GFD without oats; GFD-oats: GFD with uncontaminated oats.
